# Genetic response to metabolic fluctuations: correlation between central carbon metabolism and DNA replication in *Escherichia coli*

**DOI:** 10.1186/1475-2859-10-19

**Published:** 2011-03-31

**Authors:** Monika Maciąg, Dariusz Nowicki, Laurent Janniere, Agnieszka Szalewska-Pałasz, Grzegorz Węgrzyn

**Affiliations:** 1 Department of Molecular Biology, University of Gdańsk, Kładki 24, 80-822 Gdańsk, Poland; 2 MEGA Laboratory, Institute of System and Synthetic Biology, Génopole Campus I, 5 rue Henri Desbruères, 91000 Evry, France

## Abstract

**Background:**

Until now, the direct link between central carbon metabolism and DNA replication has been demonstrated only in *Bacillus. subtilis*. Therefore, we asked if this is a specific phenomenon, characteristic for this bacterium and perhaps for its close relatives, or a more general biological rule.

**Results:**

We found that temperature-sensitivity of mutants in particular genes coding for replication proteins could be suppressed by deletions of certain genes coding for enzymes of the central carbon metabolism. Namely, the effects of *dnaA46*(ts) mutation could be suppressed by dysfunction of *pta *or *ackA*, effects of *dnaB*(ts) by dysfunction of *pgi *or *pta*, effects of *dnaE486*(ts) by dysfunction of *tktB*, effects of *dnaG*(ts) by dysfunction of *gpmA, pta *or *ackA*, and effects of *dnaN159*(ts) by dysfunction of *pta *or *ackA*. The observed suppression effects were not caused by a decrease in bacterial growth rate.

**Conclusions:**

The genetic correlation exists between central carbon metabolism and DNA replication in the model Gram-negative bacterium, *E. coli*. This link exists at the steps of initiation and elongation of DNA replication, indicating the important global correlation between metabolic status of the cell and the events leading to cell reproduction.

## Background

When considering a bacterial cell as a microbial factory, producing various macromolecules either natural or formed due to introduction of recombinant genes, several biochemical processes must be taken into consideration. Among them, there are two basic processes ensuring that more specialized reactions (like transcription of particular genes and translation of particular mRNAs on ribosomes as well as enzyme-mediated production of various compounds) can occur. These two processes are central carbon metabolism (for a review see ref. [1]) and DNA replication (for a review see ref. [2]). The former one provides energy from nutrients, which is absolutely necessary to all life functions of cells. The latter one, although consuming cellular energy, ensures integrity of genetic material and its inheritance by daughter cells after each cell division, providing the source of information about biological structures and functions of macromolecules.

The central carbon metabolism (CCM) is generally recognized as a set of biochemical pathways devoted to transport and oxidation of main carbon sources in the cell [1]. In a model Gram-negative bacterium, *Escherichia coli*, it consists of the phosphortransferase system, glycolysis, gluconeogenesis, pentose-monophosphate bypass with Entner-Dudoroff pathway, Krebs cycle with glyoxylate bypass and the respiration chain [3]. Biochemical reactions of these pathways ensure the optimal energy production and usage in the cell at particular growth conditions, in order to keep homeostasis.

DNA replication is a process of genetic information duplication, which is necessary to equal and precise distribution of the genetic material to both daughter cells after each cell division [2]. The process of replicative DNA synthesis requires large cellular machinery, which in *E. coli *consists of DNA polymerase III holoenzyme (containing at least 10 subunits) and other essential proteins, including DnaB helicase and DnaG primase. Additional proteins (DnaA, DnaC) are required for DNA replication initiation at a specific genome region, called *oriC *[2,4].

Although it was observed previously that regulation of DNA replication may depend on bacterial cell metabolism, it was generally assumed that this dependency is indirect. For example, it might result from different availability of cellular energy and/or precursors of macromolecules [5,6] or from production of specific alarmons, like cyclic AMP (cAMP) [7,8] or guanosine tetraphosphate (ppGpp) [9-12], in response to nutritional deprivations. However, it was reported recently that DNA replication may be directly linked to central carbon metabolism, particularly glycolysis, in a model Gram-positive bacterium, *Bacillus subtilis *[13]. Namely, specific suppression of conditionally-lethal (temperature-sensitive, ts) mutations in genes coding for replication proteins (DnaE, a DNA polymerase involved in lagging strand synthesis, DnaC, a helicase - homologue of *E. coli *DnaB protein, and DnaG, the primase) by dysfunction of certain genes coding for enzymes involved in glycolysis, was observed. An indirect suppression mechanism (e.g. by slowing down of bacterial growth rate) was excluded, strongly suggesting a real link between glycolysis and DNA replication. Thus, the existence of such a link should be considered in any studies on both these processes, as well as when constructing and using biotechnological systems for efficient production of desired compounds.

Until now, the direct link between central carbon metabolism and DNA replication has been demonstrated only in *B. subtilis *[13]. Therefore, we asked if this is a specific phenomenon, characteristic for this bacterium and perhaps for its close relatives, or a more general biological rule. Since *E. coli *is both a model Gram-negative bacterium and a widely used host for production of recombinant proteins, in our studies, which were performed to answer the above question, we employed strains of this species.

## Methods

### Bacterial strains, plasmids and bacteriophages

*E. coli *strains used in this work are listed in Table 1. Plasmids and bacteriophages are described in Table 2. New bacterial strains and plasmids were constructed according to standard procedures of P1 transduction and molecular cloning, respectively [14].

**Table 1:** *E. coli *strains used in this work

Strain	Relevant characteristics	Reference or source
JJC809 (PC8)	*dnaB8*(ts) Cm^R ^F2 *leuB6 thyA47 deoC3 rps153 *l2	[21]
PC2	*dnaC(*ts*) thy leu rpsL*	[21]
PC3	*dnaG(ts) Hfr leu thy rpsL*	[22]
MG1655	F- λ- *ilvG*- *rfb*-50 *rph*-1	[23]
MG1655*dnaA*46	F- λ- *ilvG*- *rfb*-50 *rph*-1 *dnaA*46 *tna*::Tn10	[24]
DH5α	*F- φ80lacZΔ*M15 *Δ(lac*ZYA*-argF*)U169 *deoR recA1 endA1 hsdR17*(rk-, mk+) *phoA supE44 thi-1 gyr*A96 *rel*A1 λ^-^	[25]
*BW25113*	Δ*(araD-araB)567*, Δ*lacZ4787*::*rrnB*-3, λ^-^, *rph-1*, Δ*(rhaD-rhaB)568*, *hsdR514*	[26]
JW1122	Same as BW25113 but *Δicd::kan*	[27]
JW1413	Same as BW25113 but *ΔgapC::kan*	[27]
JW1666	Same as BW25113 but *ΔpykF::kan*	[27]
JW1841	Same as BW25113 but *Δzwf::kan*	[27]
JW2449	Same as BW25113 but *ΔtktB::kan*	[27]
JW3366	Same as BW25113 but *Δpck::kan*	[27]
JW3890	Same as BW25113 but *ΔtpiA::kan*	[27]
JW3974	Same as BW25113 but *ΔaceB::kan*	[27]
JW3985	Same as BW25113 but *Δpgi::kan*	[27]
JW2294	Same as BW25113 but *Δpta::kan*	[27]
JW2293	Same as BW25113 but *ΔackA::kan*	[27]
JW5173	Same as BW25113 but *ΔicdC::kan*	[27]
JW5344	Same as BW25113 but *ΔfbaB::kan*	[27]
JW0738	Same as BW25113 but *ΔgpmA::kan*	[27]
NR13339	Same as KA796 with *dnaN159(*Ts*) zid501::*Tn10	[28]
NR7651	Same as MC4100 *lacZ*104 *dnaE486(*Ts*) zae502::*Tn10	[28]
AS701	MG1655 *dnaA46 Δacn::kan*	This study, by P1 transduction from JW0114
AS702	MG1655 *dnaA46 Δicd::kan*	This study, by P1 transduction from JW1122
AS703	MG1655 *dnaA46 ΔgapC::kan*	This study, by P1 transduction from JW1413
AS704	MG1655 *dnaA46 ΔpykF::kan*	This study, by P1 transduction from JW1666
AS705	MG1655 *dnaA46 Δzwf::Kan*	This study, by P1 transduction from JW1841
AS706	MG1655 *dnaA46ΔtktB::kan*	This study, by P1 transduction from JW2449
AS707	MG1655 *dnaA46 Δpck::Kan*	This study, by P1 transduction from JW3366
AS708	MG1655 *dnaA46 ΔtpiA::Kan*	This study, by P1 transduction from JW3890
AS709	MG1655 *dnaA46 ΔaceB:Kan*	This study, by P1 transduction from JW3974
AS710	MG1655 *dna A46 Δpgi::kan*	This study, by P1 transduction from JW3985
AS711	MG1655 *dna A46 Δpta::kan*	This study, by P1 transduction from JW2294
AS712	MG1655 *dnaA46 ΔackA::kan*	This study, by P1 transduction from JW2293
AS713	MG1655 *dnaA46 ΔicdC::kan*	This study, by P1 transduction from JW5173
AS714	MG1655 *dnaA46ΔfbaB::kan*	This study, by P1 transduction from JW5344
AS715	MG1655 *dnaA46 ΔgpmA::kan*	This study, by P1 transdukcion from JW0738
AS766	MG1655 *dnaB8 Δacn::kan*	This study, by P1 transduction from JW0114
AS767	MG1655 *dnaB8 Δicd::kan*	This study, by P1 transduction from JW1122
AS768	MG1655 *dnaB8 ΔgapC::kan*	This study, by P1 transduction from JW1413
AS769	MG1655 *dnaB8 ΔpykF::kan*	This study, by P1 transduction from JW1666
AS770	MG1655 *dnaB8 Δzwf::kan*	This study, by P1 transduction from JW1841
AS771	MG1655 *dnaB8 ΔtktB::kan*	This study, by P1 transduction from JW1841
AS772	MG1655 *dnaB8 Δpck::kan*	This study, by P1 transduction from JW3366
AS773	MG1655 *dnaB8 ΔtpiA::kan*	This study, by P1 transduction from JW3890
AS774	MG1655 *dnaB8 ΔaceB::kan*	This study, by P1 transduction from JW3974
AS775	MG1655 *dnaB8 Δpgi::kan*	This study, by P1 transduction from JW3985
AS776	MG1655 *dnaB8 Δpta::kan*	This study, by P1 transduction from JW2294
AS778	MG1655 *dnaB8 ΔackA::kan*	This study, by P1 transduction from JW2293
AS779	MG1655 *dnaB8 ΔicdC::kan*	This study, by P1 transduction from JW5173
AS780	MG1655 *dnaB8 ΔfbaB::kan*	This study, by P1 transduction from JW5344
AS781	MG1655 *dnaB8 ΔgpmA::kan*	This study, by P1 transduction from JW0738
AS750	PC2 dnaC*(ts) Δacn::kan*	This study, by P1 transduction from JW0114
AS751	PC2 *dnaC(ts) Δicd::kan*	This study, by P1 transduction from JW1122
AS752	PC2 *dnaC(ts) ΔgapC::kan*	This study, by P1 transduction from JW1413
AS753	PC2 *dnaC(ts) ΔpykF::kan*	This study, by P1 transduction from JW1666
AS754	PC2 *dnaC(ts) Δzwf::kan*	This study, by P1 transduction from JW1841
AS755	PC2 *dnaC(ts) ΔtktB::kan*	This study, by P1 transduction from JW2449
AS756	PC2 *dnaC(ts) Δpck::kan*	This study, by P1 transduction from JW3366
AS757	PC2 *dnaC(ts) ΔtpiA::kan*	This study, by P1 transduction from JW3890
AS758	PC2 *dnaC(ts) ΔaceB::kan*	This study, by P1 transduction from JW3974
AS759	PC2 *dnaC(ts) Δpgi::kan*	This study, by P1 transduction from JW3985
AS760	PC2 *dnaC(ts) Δpta::kan*	This study, by P1 transduction from JW2294
AS761	PC2 *dnaC(ts) ΔackA::kan*	This study, by P1 transduction from JW2293
AS762	PC2 *dnaC(ts) ΔicdC::kan*	This study, by P1 transduction from JW5173
AS763	PC2 *dnaC(ts) ΔfbaB::kan*	This study, by P1 transduction from JW5344
AS764	PC2 *dnaC(ts) ΔgpmA::kan*	This study, by P1 transduction from JW0738
AS783	PC3 *dnaG(ts) Δacn::kan*	This study, by P1 transduction from JW0114
AS784	PC3 *dnaG(ts) Δicd::kan*	This study, by P1 transduction from JW1122
AS785	PC3 *dnaG(ts) ΔgapC::kan*	This study, by P1 transduction from JW1413
AS786	PC3 *dnaG(ts) ΔpykF::kan*	This study, by P1 transduction from JW1666
AS787	PC3 *dnaG(ts) Δzwf::kan*	This study, by P1 transduction from JW1841
AS788	PC3 *dnaG(ts) ΔtktB::kan*	This study, by P1 transduction from JW2449
AS789	PC3 *dnaG(ts) Δpck::kan*	This study, by P1 transduction from JW3366
AS790	PC3 *dnaG(ts) ΔtpiA::kan*	This study, by P1 transduction from JW3890
AS791	PC3 *dnaG(ts) ΔaceB::kan*	This study, by P1 transduction from JW3974
AS792	PC3 *dnaG(ts) Δpgi::kan*	This study, by P1 transduction from JW3985
AS793	PC3 *dnaG(ts) Δpta::kan*	This study, by P1 transduction from JW2294
AS794	PC3 *dnaG(ts) ΔackA::kan*	This study, by P1 transduction from JW2293
AS795	PC3 *dnaG(ts) ΔicdC::kan*	This study, by P1 transduction from JW7173
AS796	PC3 *dnaG(ts) ΔfbaB::kan*	This study, by P1 transduction from JW5344
AS797	PC3 *dnaG(ts) ΔgpmA::kan*	This study, by P1 transduction from JW0738
AS718	MG1655 *dnaE486 Δacn*	This study, by P1 transduction from JW0114
AS719	MG1655 *dnaE486 Δicd*	This study, by P1 transduction from JW1122
AS720	MG1655 *dnaE486 ΔgapC*	This study, by P1 transduction from JW1413
AS721	MG1655 *dnaE486 ΔpykF*	This study, by P1 transduction from JW1666
AS722	MG1655 *dnaE486 Δzwf*	This study, by P1 transduction from JW1841
AS723	MG1655 *dnaE486 ΔtktB*	This study, by P1 transduction from JW2449
AS724	MG1655 *dnaE486 Δpck*	This study, by P1 transduction from JW3366
AS725	MG1655 *dnaE486 ΔtpiA*	This study, by P1 transduction from JW3890
AS726	MG1655 *dnaE486 ΔaceB*	This study, by P1 transduction from JW3974
AS728	MG1655 *dnaE486 Δpgi*	This study, by P1 transduction from JW3985
AS729	MG1655 *dnaE486 Δpta*	This study, by P1 transduction from JW2294
AS730	MG1655 *dnaE486 ΔackA*	This study, by P1 transduction from JW2293
AS731	MG1655 *dnaE486 ΔicdC*	This study, by P1 transduction from JW5173
AS732	MG1655 *dnaE486 ΔfbaB*	This study, by P1 transduction from JW5344
AS733	MG1655 *dnaE486 ΔgpmA*	This study, by P1 transduction from JW0738
AS734	MG1655 *dnaN159 ΔacnB::kan*	This study, by P1 transduction from JW0114
AS735	MG1655 *dnaN159 Δicd::kan*	This study, by P1 transduction from JW1122
AS736	MG1655 *dnaN159 ΔgapC::kan*	This study, by P1 transduction from JW1413
AS737	MG1655 *dnaN159 ΔpykF::kan*	This study, by P1 transduction from JW1666
AS738	MG1655 *dnaN159 Δzwf::kan*	This study, by P1 transduction from JW1841
AS739	MG1655 *dnaN159 ΔtktB::kan*	This study, by P1 transduction from JW2449
AS740	MG1655 *dnaN159 Δpck::kan*	This study, by P1 transduction from JW3366
AS741	MG1655 *dnaN159 ΔtpiA::kan*	This study, by P1 transduction from JW3890
AS742	MG1655 *dnaN159 ΔaceB::kan*	This study, by P1 transduction from JW3974
AS743	MG1655 *dnaN159 Δpgi::kan*	This study, by P1 transduction from JW3985
AS744	MG1655 *dnaN159 Δpta::kan*	This study, by P1 transduction from JW2294
AS745	MG1655 *dnaN159 ΔackA::kan*	This study, by P1 transduction from JW2293
AS746	MG1655 *dnaN159 ΔicdC::kan*	This study, by P1 transduction from JW5173
AS747	MG1655 *dnaN159 ΔfbaB::kan*	This study, by P1 transduction from JW5344
AS748	MG1655 *dnaN159 ΔgpmA::kan*	This study, by P1 transduction from JW0738
AS700	MG1655 *dnaN159 zid501::*Tn10	This study, by P1 transduction from NR13339
AS717	MG1655 *dnaE486 zae502::*Tn10	This study, by P1 transduction from NR7651
AS765	MG1655 *dnaB8(ts) cmR*	This study, by P1 transduction from JJC809

**Table 2:** Plasmids employed and constructed in this study

Plasmid	Relevant characteristics	Reference
pBAD24	Ori pBR322; *bla+ *P_BAD_	[29]
pAS101	pBAD24 bearing the *ackA *gene under of pBAD control	This study, by cloning of a PCR amplified fragment of *E. coli *MG1655 chromosome, obtained with primers ackaF and ackaR (Table 3), into the SmaI side of pBAD24
pAS102	pBAD24 bearing the *pgi *gene under of pBAD control	This study, by cloning of a PCR amplified fragment of *E. coli *MG1655 chromosome fragment obtained with primers pgiF and pgiR (Table 3), into the SmaI side of pBAD24
pAS103	pBAD24 bearing the *fbaB *gene under of pBAD control	This study, by cloning of a PCR amplified fragment of *E. coli *MG1655 chromosome fragment obtained with primers fbabF and fbabR (Table 3), into the KpnI side of pBAD24
pAS104	pBAD24 bearing the *tktB *gene under of pBAD control	This study, by cloning of a PCR amplified fragment of *E. coli *MG1655 chromosome fragment obtained with primers tktbF and tktbR (Table 3), into the KpnI side of pBAD24
pAS105	pBAD24 bearing the *pta *gene under of pBAD control	This study, by cloning of a PCR amplified fragment of *E. coli *MG1655 chromosome fragment obtained with primers ptaF and ptaR (Table 3), into the KpnI side of pBAD24
pAS106	pBAD24 bearing the *gpm *gene under of pBAD control	This study, by cloning of a PCR amplified fragment of *E. coli *MG1655 chromosome fragment obtained with primers gpmaF and gpmaR (Table 3), into the KpnI side of pBAD24
pAS107	pBAD24 bearing the *aceB *gene under of pBAD control	This study by cloning of a PCR amplified fragment of *E. coli *MG1655 chromosome fragment obtained with primers acebF and acebR (Table 3), into the KpnI side of pBAD24

### Oligonucleotides

Oligunucleotides are described in Table 3.

**Table 3:** Oligonucleotides used for cloning

Primer name	Primer sequence (5'>3')	Tm °C	Restriction enzyme site
ackaF	GGCCCGGGATGTCGAGTAAGTTAG	58.0	SmaI
ackaR	TGGCAAGCTTACATTCAGGCAGTCAGGCGGCTCG	60.0	HindIII
gpmaF	CCGGGTACCATGGCTGTAACTAAGCTGGTTCTG	66.9	KpnI
gpmaR	CGCGGTCGACTTACTTCGCTTTACCCTGG	65.7	SalI
fbabF	TCCGGTACCATGACAGATATTGCGCAGTTGCTTG	65.6	KpnI
fbabR	GGCCGTCGACTCAGGCGATAGTAATTTTGC	64.4	SalI
pgiF	GCCCGGGATGAAAAACATCAATCCAACGCAGACC	66.8	SmaI
pgiR	CGGAAGCTTTGATTAACCGCGCCACGCTTTATAG	65.6	HindIII
ptaF	CGGAGGAGGTACCATGTCCCGTATTATTATG	63.0	KpnI
ptaR	GACGAAGCTTAGATTACTGCTGCTGTGCAGAC	64.4	HindIII
tktbF	CGGAGGGTACCATGTCCCGAAAAGACCTTG	54.0	KpnI
tktbR	GCGCAAGCTTTCAGGCACCTTTCACTCCC	57.0	HindIII
acebF	GAGCGGTACCATGACTGAACAGGCAACAACAAC	58.0	KpnI
acebR	TGTGTCGACTTACGCTAACAGGCGGTAGCCTGG	58.0	SalI

Sequences of particular oligonucleotides recognized by restriction enzymes listed in corresponding row are underlined.

### Growth conditions

Luria -Bertani (LB) medium, and minimal media M9 and MM, were used [14]. Solid media contained 1.5% of bacteriological agar. For liquid cultures, bacteria were grown in various media in shake flasks, with aeration (by shaking). Overnight cultures were diluted in LB and grown to OD_600 _= 0.3. Then, 100 μl of the culture or its dilution was plated on solid media. The plates were then incubated at indicated temperatures for indicated time. CFU (colony forming units) were calculated from plates where colony number was between 100 and 1000.

## Results

We have employed six *E. coli *temperature-sensitive mutants in following genes coding for proteins necessary for chromosomal DNA replication: *dnaA *(coding for the replication initiator protein that binds to the *oriC *region and forms a specific nucleoprotein structure; this is the first step in the DNA replication initiation), *dnaB *(coding for the main DNA helicase, the enzyme necessary to melt DNA during the replication process), *dnaC *(coding for the protein which delivers DnaB helicase to the DnaA protein bound to *oriC*), *dnaE *(coding for the α subunit of DNA polymerase III, the catalytic subunit of this enzyme), *dnaG *(coding for primase, an enzyme necessary to synthesize RNA primers during DNA replication) and *dnaN *(coding for the β subunit of DNA polymerase III, a protein forming the sliding clamp and allowing DNA polymerase III to be kept on the template DNA strand when synthesizing new polynucleotide strand) [for more detailed information on these genes and their products, see ref. 2]. These mutants are described in Table 1.

**Figure 1:**
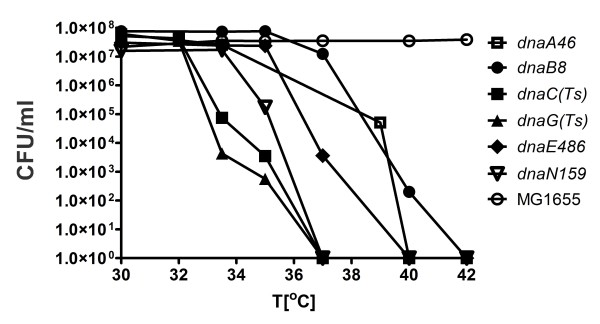
**Temperature-sensitivity profiles of wild type and mutant strains**. The growth and plating conditions were as described in Methods.

To test whether mutations (particularly deletion-insertion mutations) in genes coding for enzymes from central carbon metabolism (CCM) may suppress temperature sensitivity of the replication mutants, we have determined the sensitivity profiles of all tested conditionally lethal mutants. This was necessary to chose temperatures that severely restricted growth of mutant cells, however, which still allowed observing some viability of tested strains; otherwise detection of any suppression would be impossible, as observed in the *B. subtilis *study [13]. The profiles of temperature-sensitivity of *dnaA, dnaB, dnaC, dnaE, dnaG *and *dnaN *mutants in LB medium are shown in Figure 1.

**Figure 2:**
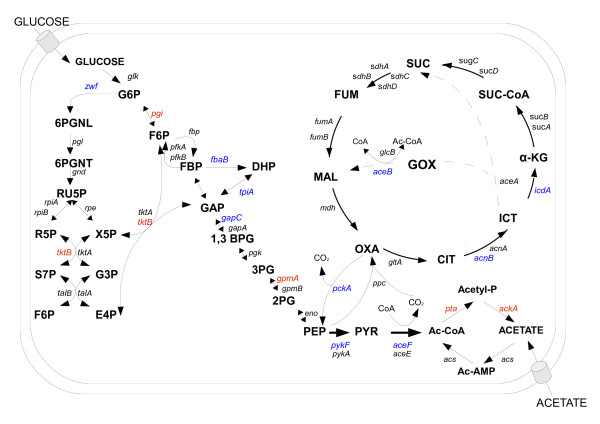
**A scheme for CCM including main pathways - glycolysis/gluconeogenesis, penthaphosphate pathway, citrate cycle, overflow pathway**. Mutants tested in this work are indicated by following colours: blue - non suppressor mutants, red - suppressors of replication genes mutants. Metabolites abbreviations: 1,3-BGP, 1,3-biphosphoglycerate; 2PG, 2-phophoglycerate; 3PG, 3-phosphoglycerate; 6PGLN, 6-phosphoglucono-δ-lactone; 6PGNT, 6-phophogluconate; GLC, glucose; G6P, glucose-6-phosphate; F6P, fructose-6-phosphate; FUM, fumarate; MAL, malate; OXA, oxaloacetate PBP, fructose-1,6-biphosphate; DHAP, dihydroxyacetone phosphate; GAP, glyceraldehyde 3-phosphate; PEP, phosphoenolpyruvate; PYR, pyruvate; Ru5P, ribulose-5-phosphate; R5P, ribose-5-phosphate; S7P, sedoheptulose-7-phosphate; E4P, erythrose-4-phosphate; Ac-CoA, acetyl coenzyme A; Ac-P, acetyl phosphate; Ac-AMP, acetyl-AMP; CIT, citrate; ICT, isocitrate; GOX, glyoxylate; α-KG, α-ketoglutarate; SUC-CoA, succinyl-coenzyme A; SUC, succinate; Xu5P, xylulose-5-phosphate.

A series of double mutants, bearing mutations in one of the replication genes and in one of genes coding for CCM enzyme, has been constructed by P1 transduction (Table 1). For these constructions, deletion-insertion mutants in following genes were employed: *gapC, pykF, tpiA, pgi, fbaB, gpmA, pck, zwf, tktB, pta, ackA, aceB, acnB*, and *icd*. Enzymes encoded by these genes are listed in Table 4, and locations (in particular biochemical pathways) of reactions catalyzed by them are marked on the scheme depicting the central carbon metabolism in *E. coli *(Figure 2).

**Table 4:** Enzymes of CCM, whose genes were tested in this study

EC number	Name	Gene	Pathway
EC 1.2.1.9	Glyceraldehyde-3-phosphate dehydrogenase	*gapC*	glycolysis/gluconeogenesis
EC 2.7.1.40	Pyruvate kinase	*pykF*	
EC 5.3.1.1	Triose-phosphate isomerase	*tpiA*	
EC 5.3.1.9	Glucose-6-phosphate isomerase	*pgi*	
EC 4.1.2.13	Fructose-bisphosphate aldolase	*fbaB*	
EC 5.4.2.1	Phosphoglyceromutase	*gpmA*	
EC 4.1.1.49	Phosphoenolpyruvate carboxykinase (ATP)	*pckA*	

EC 1.1.1.49	Glucose-6-phosphate 1-dehydrogenase	*zwf*	pentose phosphate pathway
EC 2.2.1.1	Transketolase B	*tktB*	

EC 2.3.1.8	Phosphate acetyltransferase	*pta*	overflow pathway
EC 2.7.2.1	Acetate kinase	*ackA*	
EC 2.3.1.12	Dihydrolipoyllysine-residue acetyltransferase	*aceF*	

EC 2.3.3.9	Malate synthase	*aceB*	citrate cycle (TCA cycle)
EC 4.2.1.3	Aconitate hydratase	*acnB*	
EC 1.1.1.42	Isocitrate dehydrogenase, specific for NADP+	*icdA*	
-	Conserved hypothetical protein (pseudogene)	*icdC*	

We have tested whether mutations in the CCM genes can suppress temperature sensitivity of bacteria caused by mutations in the replication genes. In this test, bacteria were plated at sublethal temperatures (i.e. temperatures causing a decrease in the efficiency of plating for several orders of magnitude, but still allowing survival of a small fraction of mutant cells), selected on the basis of temperature-sensitivity profiles determined as shown in Figure 1 (in control experiments, the temperature permissive to all strains, 30°C, was used). These following sublethal temperatures were chosen for particular replication mutants: 39°C for *dnaA46*(ts), 41°C for *dnaB8*(ts), 35°C for *dnaC*(ts), 36.5°C for *dnaE486*(ts), 34°C for *dnaG*(ts) and 37.5°C for *dnaN159*(ts).

**Figure 3:**
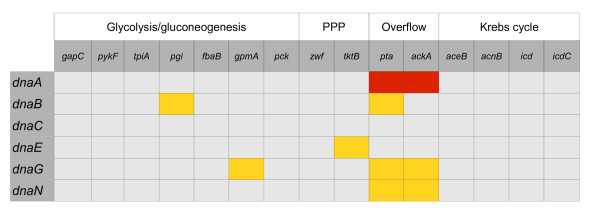
**Suppression pattern of double mutants in CCM and replication genes**. Red - full suppression, yellow - incomplete suppression. Suppressions were observed in sublethal temperatures.

**Figure 4:**
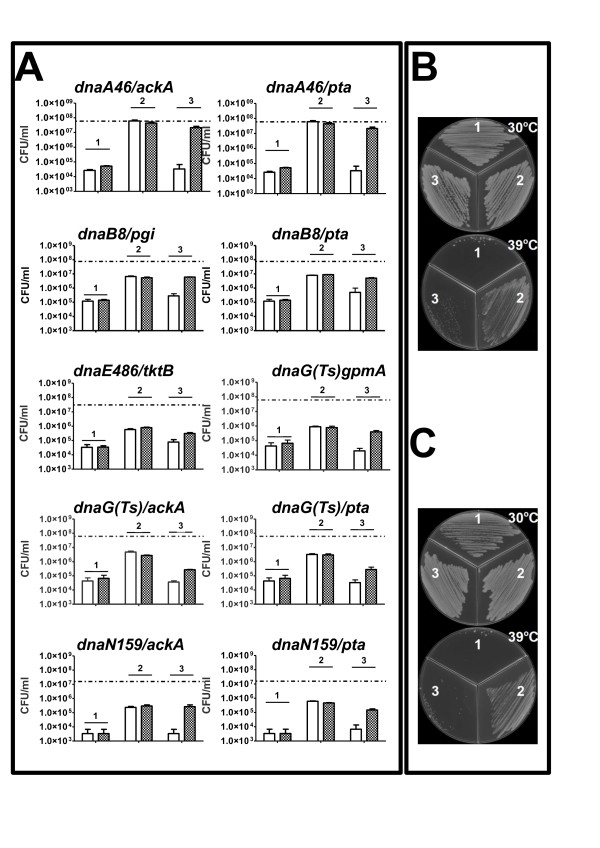
**Complementation of suppression phenotypes in double replication/CCM mutants by the overproduction of the metabolic enzymes**. The experiments were performed in sublethal temperatures (relevant for each strain). Mutations as indicated above the graphs were employed. Panel A. Bacterial growth measured in CFU. Empty columns - growth in the presence of 0.2% arabinose, shaded columns - growth in the presence of 0.1% glucose. Efficiencies of plating (CFU/ml) of the replication mutants at 30°C are indicated by a dashed line at each graph. Panel B and C. The growth of temperature sensitive dnaA46-derivatives in permissive and sublethal temperature. B - *dnaA46Δpta*, C - *dnaA46ΔackA*. Panels A, B and C. 1 - temperature-sensitive replication mutants, 2 - double mutants in replication and CCM genes, 3 - double mutants in replication and CCM genes complemented with the relevant metabolic gene under the control of arabinose-inducible pBAD promoter.

We found no specific suppression (i.e. suppression which could be reversed by plasmid-mediated expression of the wild-type CCM gene whose defective allele resulted in temperature-tolerance of otherwise temperature-sensitive replication mutant) of the effects of *dnaC*(ts) mutation by any tested dysfunction in the CCM genes (Figure 3). However, interestingly, efficiency of plating of *dnaA46*(ts), *dnaB8*(ts), *dnaE486*(ts), *dnaG*(ts) and *dnaN159*(ts) mutants could be increased by at least one order of magnitude (often considerably more) at sublethal temperatures in the presence of particular mutations in genes coding for enzymes from CCM (Figure 3). The effects of *dnaA46*(ts) mutation could be suppressed by dysfunction of *pta *or *ackA*, effects of *dnaB8*(ts) by dysfunction of *pgi *or *pta*, effects of *dnaE486*(ts) by dysfunction of *tktB*, effects of *dnaG*(ts) by dysfunction of *gpmA, pta *or *ackA*, and effects of *dnaN159*(ts) by dysfunction of *pta *or *ackA*. Most of the suppression phenomena were not complete, i.e. the efficiency of survival of the ts mutants in the sublethal temperature was between 1 and 10% of that in the permissive temperature, though still it was 10 to 100 times higher than that of the ts mutant without suppressor mutation at the sublethal temperature (Figure 4). This correlates with the previous findings on the *B. subtilis *model [13]. Interestingly, the only exceptions were *dnaA46 *suppressors, restoring 100% growth relative to that under permissive conditions. It is worth noting that *dnaA *mutants of *B. subtilis *were not tested in the previous work, mentioned above [13].

To test whether suppressions depicted in Figure 3 were specific, plasmids bearing wild-type copies of disrupted metabolic genes (Table 2) have been introduced into cells of the double mutants. The wild-type alleles were under control of the pBAD promoter, which could be stimulated by addition of L-arabinose into growth medium. We found that for *dnaA46*(ts), *dnaB8*(ts), *dnaE486*(ts), *dnaG*(ts) and *dnaN159*(ts) hosts, expression of appropriate wild-type allele of CCM gene reversed effects of temperature sensitivity suppression by the corresponding mutant allele (Figure 4). Therefore, we conclude that the suppression effects depicted in Figure 3 are specific for certain mutations.

**Figure 5:**
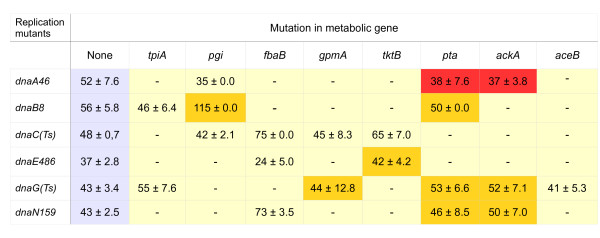
**Generation times of double mutants in replication and CCM genes**. Bacteria were grown at 30°C in LB and doubling time (values presented in the boxes ± SD) was assessed in the exponential growth phase. The doubling time for the wild-type strain (MG1655) was 48 ± 0.7 min. The colors represent genotypes in which suppressions were observed at sublethal temperatures (red - full suppression, yellow - incomplete suppression). Dash - the generation time was not determined.

We asked whether the suppression of temperature sensitivity of mutants in the replication genes by dysfunction of particular genes coding for CCM enzymes could be caused by decreased growth rates of double mutants. This question was substantiated by the fact that DNA replication regulation is known to be dependent on bacterial growth rate [2]. However, we found that although in most cases (excluding the *dnaA46 *mutants) at 30°C the growth rates of the double mutants revealing suppression of the temperature sensitivity were lower than in wild-type bacteria, a similar or lower decrease in the growth rate was observed also in double mutants which did not suppress the temperature sensitivity (Figure 5). Therefore, we conclude that the observed suppression effects could not be caused simply by a decrease in bacterial growth rate.

We have also tested whether the suppression can be caused by growth of the replication mutants in media containing various carbon sources, which also allow for different growth rates. Therefore, we have plated *dnaA46*(ts), *dnaB8*(ts), *dnaC*(ts), *dnaE486*(ts), *dnaG*(ts) and *dnaN159*(ts) mutants on plates containing a minimal medium supplemented with various carbon sources: glucose, glycerol, maleic acid or sodium acetate. However, in these experiments, we did not observe any improvement in viability of these mutants at the sublethal temperatures (data not shown). These results corroborate the results of experiments with growth rate measurement, and support our conclusion that the suppression of temperature sensitivity of the replication mutants cannot be explained by lower growth rates of bacteria.

## Discussion

The approach to understand cellular processes as a network of complex relations becomes more appreciated only nowadays. Two major processes responsible for maintenance and reproduction of the cell (i.e. energy metabolism and DNA replication) were studied mostly independently until recently. A direct link between DNA replication and central carbon metabolism (CCM) has been demonstrated solely for one species of Gram-positive bacterium, *B. subtilis *[13]. This finding was a breakthrough in considering these processes as interrelated. Thus, it was crucial to address the question whether such a phenomenon occurs only in the specific strain or it is more general. Here we present evidence that such a link exists also in *E. coli*, a model Gram-negative bacterium.

Despite the general similarity, there are important differences between suppression of effects of mutations in replication genes by dysfunction of genes coding for enzymes of CCM in *E. coli *and *B. subtilis*. According to previous report [13], in *B. subtilis*, the temperature-sensitivity suppression was detected for only three genes: *dnaE, dnaC *(an equivalent of the *E. coli dnaB *gene, coding for helicase) and *dnaG*. Temperature-sensitive mutants in these genes could grow at elevated temperatures in the presence of additional mutations in *gapA*, *pgk*, *pgm, eno *or *pykA*. These five genes code for enzymes acting at the late stages of glycolysis and gluconeogenesis. In *E. coli*, we were able to observe suppression of effects of temperature-sensitive mutations not only in *dnaE*, *dnaB *and *dnaG *genes (like in *B. subtilis*), but also in *dnaN *and - perhaps the most surprisingly - in *dnaA*. Moreover, growth at sublethal temperatures of these mutants was observed under conditions of a lack of enzymes involved not only in glycolysis and gluconeogenesis (*pgi *and *gpmA*), but also in other regimens of CCM, namely the pentose phosphate pathway (*tktB *gene) and the overflow pathway (*pta *and *ackA *genes). This suggests that in *E. coli *the link between DNA replication and CCM may be broader than in *B. subtilis*. Alternatively, the observed differences might result from a partial exploration of a complex system (only some replication and metabolic genes were tested due to technical reasons, namely unavailability of viable mutants).

For *B. subtilis*, the target of the regulation by metabolic-related signals was shown to be mostly the elongation of the DNA replication process, though some suppressed replication mutations affected also replication initiation [13]. In *E. coli*, the evidence presented here shows the link between CCM and replication elongation (represented by enzymes involved in the replication complex), and initiation. One of indispensable regulators of the latter process in *E. coli *is DnaA protein [15,4]. Thus, the finding of the suppression of *dnaA46*(ts) conditionally-lethal phenotype by mutants in genes involved in CCM suggests the presence of as yet unidentified correlation. Moreover, the observed suppression was complete (100% survival at sublethal temperature relative to survival at permissive temperature), contrary to those noted for other mutants in replication genes. Both suppressors of the *dnaA46*(ts) phenotype map in the overflow pathway of CCM. This and the presence of the suppressors in genes of enzymes from other pathways beside glycolysis in *E. coli *could be explained by (i) partial exploration of the coupling system, (ii) the differences in the replication complexes in *E. coli *and *B. subtilis*, and/or (iii) different lifestyles and nutrient requirements of these bacterial species. *E. coli*, during its life cycle, may be exposed to the abrupt changes in the nutrient availability (the "feast-famine" scenario), which requires a more strict regulation, linking energy turnover and DNA replication, thus, it may profit from more metabolic sensors. Similarly to *B. subtilis*, the suppression observed in *E. coli *was not caused by a decrease in the growth rate. Moreover, the increase in the doubling time of replication mutants (by growth on the minimal media containing various carbon sources, including very poor ones, like maleic acid or acetate) did not improve their viability at sublethal temperatures.

The proposed mechanism of the regulation of DNA replication by CCM in *B. subtilis *involves a putative metabolic linker which can cause conformational changes in replication proteins to modulate replisome properties [13]. This hypothesis may be supported by the role of acetyl phosphate which can accumulate in the overflow pathway mutants. Acetyl phosphate has been proposed to function as a global signal that fits into various two-compound systems [16,17]. This may require the second, as yet unknown, protein modulating replication proteins, or the mechanism can rely on autophosphorylation. The role of acetyl phosphate in protein folding and stability has been proposed as well [18]. In this light it is interesting that AckA and Pta reduce the production of double-stranded breaks in DNA [19]. Moreover, DiaA, a DnaA-binding protein, contains a SIS motif that might bind phosphosugars [20]. These facts may provide a start point to further works on understanding the link between CCM and DNA replication.

It is worth noting that since we have used deletion-insertion mutants in genes coding for CCM enzymes, the suppressions of the temperature-sensitivity phenotypes of the replication mutants cannot be explained by direct protein-protein interactions. Indeed, numerous and large-scale interactions between replication proteins and CCM enzymes seemed unlikely, which led us to use a set of deletion mutants in tested genes. On the other hand, the use of such mutants ensured that particular enzymatic functions were absent in mutant cells, which excluded potential problems with putative partial inactivation of CCM enzymes caused by point mutations.

One should also take into consideration a possibility that changes in chemical composition of the cells caused by a lack of particular CCM enzymes might alleviate temperature sensitivity of mutants in genes coding for replication proteins. In fact, we cannot exclude that increased concentrations of some substances that accumulate due to metabolic blocks at certain steps of CCM might stabilize the temperature-sensitive replication proteins and allow them to function at higher temperatures. If so, CCM could have no effects on wild-type replication proteins and the DNA replication process in wild-type cells. However, to accept such a hypothesis it would be necessary to assume that there are at least several compounds (metabolites) able to interact specifically with several different temperature-sensitive variants of the replication proteins, resulting in their stabilization at elevated temperatures. Although still possible, such a scenario seems unlikely, therefore, we prefer the hypothesis that there is a link between CCM and DNA replication in bacterial cells.

## Conclusions

We show the genetic correlation between central carbon metabolism and DNA replication in the model Gram-negative bacterium, *E. coli*. Therefore, one might suggest that the existence of such a link is a general phenomenon rather than an event occurring very specifically in a small group of organisms. This link exists at the steps of initiation and elongation of DNA replication, indicating the important global correlation between metabolic status of the cell and the events leading to cell reproduction.

## List of abbreviations

CFU: colony forming unit; CCM: central carbon metabolism; PPP: pentose phosphate pathway; ts: temperature-sensitivity.

## Competing interests

The authors declare that they have no competing interests.

## Authors' contributions

MM and DN performed all experiments. LJ was the initiator of the project and contributed to experimental design and data analysis. ASP supervised experiments and participated in preparation of the manuscript. GW was a project leader, supervised the work and drafted the manuscript. All authors read and approved the final manuscript.
